# Transcriptator: An Automated Computational Pipeline to Annotate Assembled Reads and Identify Non Coding RNA

**DOI:** 10.1371/journal.pone.0140268

**Published:** 2015-11-18

**Authors:** Kumar Parijat Tripathi, Daniela Evangelista, Antonio Zuccaro, Mario Rosario Guarracino

**Affiliations:** Laboratory for Genomics, Transcriptomics and Proteomics (LAB-GTP), High Performance Computing and Networking Institute (ICAR), National Research Council of Italy (CNR), Via Pietro Castellino, 111, Napoli, Italy; Huazhong University of Science and Technology, CHINA

## Abstract

RNA-seq is a new tool to measure RNA transcript counts, using high-throughput sequencing at an extraordinary accuracy. It provides quantitative means to explore the transcriptome of an organism of interest. However, interpreting this extremely large data into biological knowledge is a problem, and biologist-friendly tools are lacking. In our lab, we developed Transcriptator, a web application based on a computational Python pipeline with a user-friendly Java interface. This pipeline uses the web services available for BLAST (Basis Local Search Alignment Tool), QuickGO and DAVID (Database for Annotation, Visualization and Integrated Discovery) tools. It offers a report on statistical analysis of functional and Gene Ontology (GO) annotation’s enrichment. It helps users to identify enriched biological themes, particularly GO terms, pathways, domains, gene/proteins features and protein—protein interactions related informations. It clusters the transcripts based on functional annotations and generates a tabular report for functional and gene ontology annotations for each submitted transcript to the web server. The implementation of QuickGo web-services in our pipeline enable the users to carry out GO-Slim analysis, whereas the integration of PORTRAIT (Prediction of transcriptomic non coding RNA (ncRNA) by ab initio methods) helps to identify the non coding RNAs and their regulatory role in transcriptome. In summary, Transcriptator is a useful software for both NGS and array data. It helps the users to characterize the de-novo assembled reads, obtained from NGS experiments for non-referenced organisms, while it also performs the functional enrichment analysis of differentially expressed transcripts/genes for both RNA-seq and micro-array experiments. It generates easy to read tables and interactive charts for better understanding of the data. The pipeline is modular in nature, and provides an opportunity to add new plugins in the future. Web application is freely available at: http://www-labgtp.na.icar.cnr.it/Transcriptator

## Introduction

The advent of new technologies in transcriptome studies, such as RNA-seq and micro-array, changes the face of traditional biological research approaches. Instead of studying one or more genes at a time, researchers are now able to measure simultaneously the genome wide changes and the regulation of genes under any given experimental condition. There are two major tasks are involved, first is to annotate even tens of thousands of assembled transcripts, produced by RNA-seq experiments for an organism which does not have reference transcriptome available. The second task, requires to analyze the significant functional behavior of up to few thousands of differentially expressed genes. Furthermore, with the arrival of new generation sequencing technologies and its usage, it is now possible to determine the transcription of non coding RNAs.

There is a wide array of methodologies to computationally reconstruct the transcript structure and quantify it from reads [1]. However, interpreting this extremely large data into biological knowledge is still a challenging and daunting task. For this reason, a large number of functional annotation pipelines and databases, such as DAVID [2], QuickGO [3], ESTExplorer [4], FastAnnotator [5] and other methods [6], were independently developed to address the challenge of functional annotation of the large gene list coming out from RNA-seq experiments. Both DAVID and QuickGO are very comprehensive databases and provide putative functional and gene ontological term annotations for a given set of transcripts, based on sequence similarity to known genes. These are useful tools for understanding the biological inference of transcriptional response, as well as newly explored sequences. Despite their complex and well documented functionalities, both DAVID and QuickGO usually require many manual steps that are often not easy to implement for biologists who are unfamiliar with command line procedures. Researchers also developed web tools such as FASTAnnotator and ESTExplorer. While the first performs the GO term, enzyme and domain annotations on transcripts, the second pipeline is specifically designed for EST analysis that includes the cleaning, assembly, clustering and functional annotation of ESTs. These analyses are not comprehensive, as they do not include annotations for pathways, protein-protein interactions and other functional information. Furthermore, they do not provide enrichment analysis for the functional annotation terms. Such large plethora of annotation tools and pipelines makes it difficult for the end users to decide the most suitable enrichment tool to analyze their dataset [7]. Therefore, there is a need of a computational pipeline, with a user friendly interface, which effectively translates transcriptomics data from RNA-seq or micro-array experiments into biological interpretations. To achieve this purpose, we have developed Transcriptator. There are three built-in applications in Transcriptator pipeline, the first application allows the user to functionally annotate the assembled reads dataset obtained from RNA-seq experiment in a fasta format. It uses comparative genomics approach to determine closest protein hits for the differentially expressed transcripts. Later, it carries out the functional and GO enrichment as well as clustering analysis of the expression profiles. This mode is helpful in determining the functionalities associated to differentially expressed transcripts from non model organisms, which lacks the referenced genome, and for which reads are de-novo assembled. In the second application, the pipeline only requires the id’s dataset such as Uniprot Accession, Ensembl Gene, Affymetrix 3Prime IVT, Entrez Gene and Agilent to carry out the functional annotation, enrichment and clustering analysis of the differentially expressed genes. It is useful for micro-array data and reference model organisms. The third application of Transcriptator carries out non coding RNA prediction within the given transcript’s dataset provided in a fasta formatted file. This helps the users to identify the non coding transcripts within the transcriptomics data under different experimental conditions and helps in inferring their most possible role in gene regulation. Our pipeline carries out automated BLAST (Basic Local Alignment Search Tool) [8] run on the Swiss-Prot and UniProt-TrEMBL databases [9] to find the most similar genes/proteins for the assembled transcripts. Then, functional and gene ontology annotations are carried out by QuickGo [10] (only in case of UNIPROT ACCESSION id’s) and DAVID [11] web-services. PORTRAIT [12], a Support Vector Machine (SVM) [13] based software is integrated in the pipeline to detect non-coding RNA in a transcriptomic data.

The advantages of our pipeline are as follows: i) it is very easy to use and informative in nature; ii) it produces functional as well as gene ontological annotation for the given transcripts; iii) it integrates the results from well established DAVID and QuickGO tools through web services; iv) pipeline also provides a plethora of information about enriched pathways such as KEGG [14], Panther [15], BioCarta (http://www.biocarta.com), UniProt and SwissProt features; v) it offers a report on statistical analysis of GO enrichment and enables a biologist to identify enriched biological themes, particularly GO terms related to biological process, molecular functions and cellular locations; vi) it also provides information about the SMART [16], Panther [15], Prosite [17], Prodom [18], PFAM [19] and InterPro [20] domains along with protein interactions such as Mint [21], Bind [22] for the annotated transcripts; vii) it helps the users to obtain information about the coding as well as non coding RNA in their transcriptomic data and its role in the regulation of transcriptional response, leading to major biological changes in cellular development and metabolism. [23–28].

## Materials and Methods

### System Architecture of Transcriptator Pipeline

**Fig 1:**
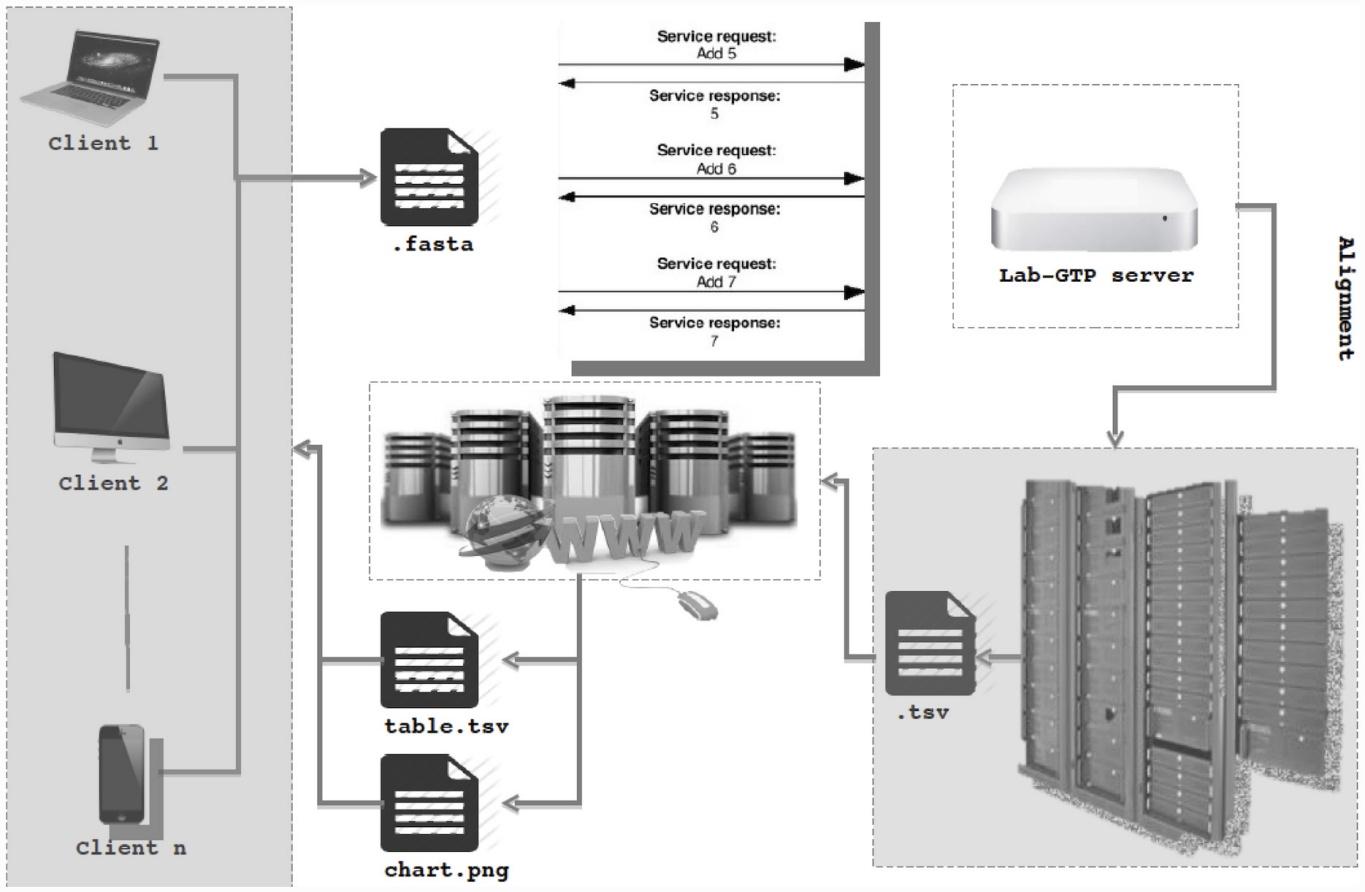
System architecture of Transcriptator Pipeline.

Trancriptator pipeline consists of three major components: (i) BLAST analysis, (ii) Gene ontology and functional annotation, retrieval and statistical analysis of the data, (iii) PORTRAIT analysis section for non coding RNA prediction. It requires various levels of computational hardware (Fig 1). This pipeline is embedded in web application written in Java and Python scripts. The front end user interface of Transcriptator is installed on LAB-GTP server. It helps the user’s to submit their queries using our web application interface. The core engine of the pipeline is written in Python, it comprises of the BLAST analysis as well as different web-services for functional annotation analysis from publicly available databases such as DAVID and QuickGO. The core engine is locally installed on a interomics cluster which is connected to LAB-GTP server. For BLAST analysis, ncbi-blast.2.2.23 stand alone package is installed on the cluster. SwissProt and UniProt-trEMBL databases (http://www.uniprot.org/) are also installed for BLAST run. DAVID and Quick-GO web-services are installed on the cluster for the faster processing of results. The query of FASTA sequence datasets provided through web application on our web server is directly transferred to our interomics cluster. Local BLAST analysis is carried out on the local cluster implying BLAST X run on locally installed SwissProt and UniProt databases. BLAST results are analyzed and top proteins hits id’s are used as input for DAVID and QUICK-GO web-services to retrieve functional and gene ontological annotations. The retrieved data are processed using statistical analysis section of Transcriptator pipeline core engine. We implemented an algorithm called PORTRAIT to predict non coding RNA’s in transcriptomic sequences obtained from various experiments. The results are provided in the form of graphs and tabular reports, and transferred to the LAB-GTP web server again. From the server, user can access to the information by using the job ID′s provided by the server.

### Pipeline implementation

**Fig 2:**
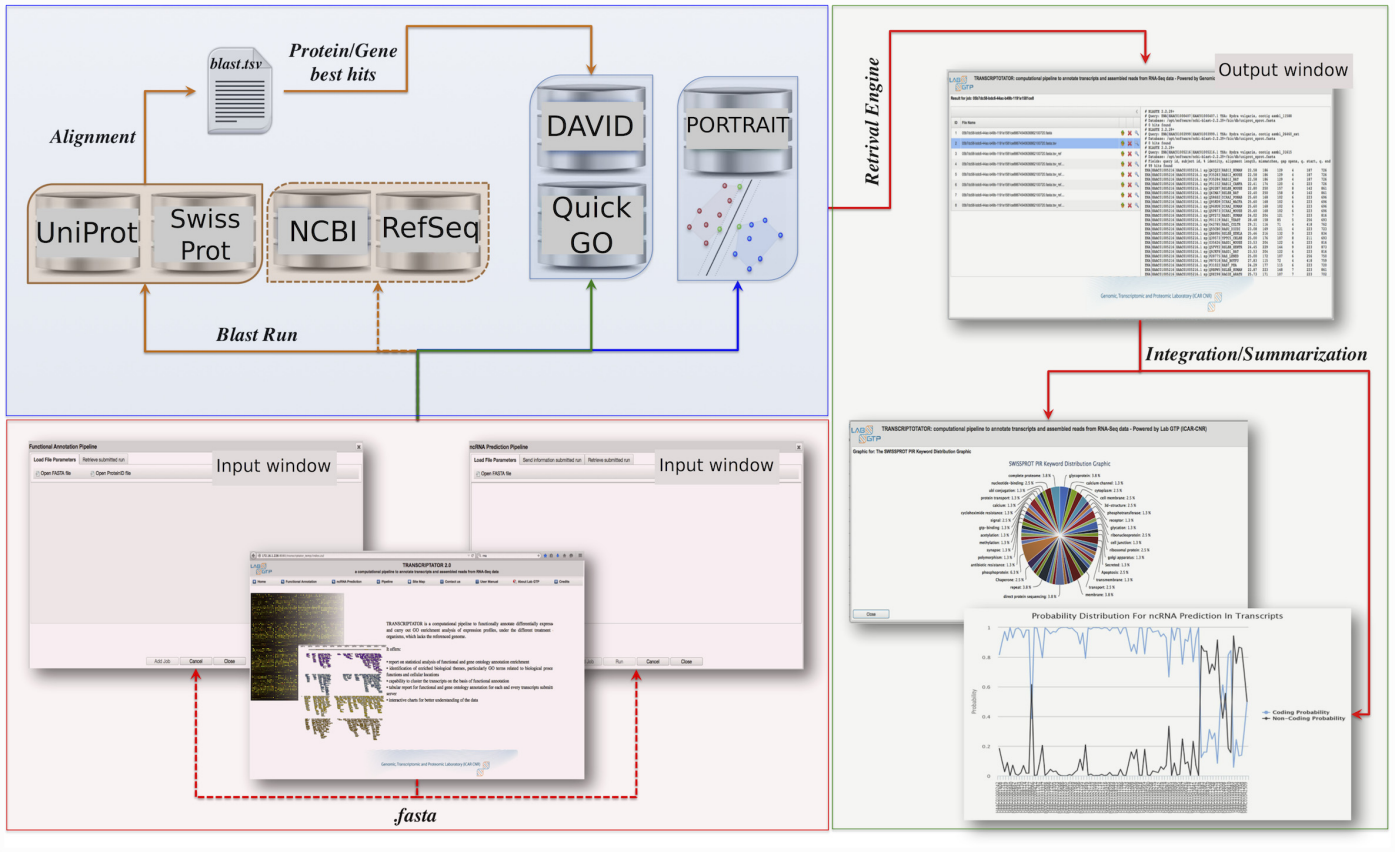
Transcriptator pipeline: the lower panel boxes respectively show the input/output of the web interface, whereas the upper panel represents the steps of the Transcriptator engine.

The Transcriptator pipeline (Fig 2) is written in Python, bash and R scripts. It implements the web services available for DAVID and QuickGO tools. For DAVID web-services, it utilizes the available Python client source code. The Python client use the light-weight soap client suds-0.4 module for DAVID web-services [https://pypi.python.org /pypi/suds]. For QuickGO web-services, BioServices Python package is used in the pipeline. It provides access to QuickGO and a framework to easily implement web service wrappers (based on WSDL/SOAP or REST protocols). In this pipeline, the annotation process for transcripts sequences comprises of four main parts: (i) finding the best hit in locally installed SwissProt and UniProt-Trembl database; (ii) assignment of functional annotation and gene ontology terms and their enrichment from DAVID; (iii) assignment of GO Slim terms and their analysis from QuickGO; (iv) integration and summarization of retrieved results from DAVID and QuickGO web services. In case of ids dataset, the BLAST step is skipped and the rest three steps are carried out. Transcriptator runs the first step of BLAST search on the local cluster, only in case of sequence input data. The second and third steps of the pipeline simultaneously runs to accelerate the annotation procedure. The last step retrieves the results, processes them and generates the statistical reports in the form of tables and charts. The non coding RNA prediction steps is carried out as separate application of our pipeline. To add this functionality, we integrates the PORTRAIT, a Support Vector Machine (SVM) based software our pipeline.

#### Identification of best hits

BLASTx program is used (with threshold E-value 0.001) to identify the best hits for query sequences on locally installed SwissProt and UniProt-trEMBL databases (http://www.uniprot.org/). The main goal of the first step is to find the similar sequences within SwissProt and UniProt-trEMBL databases for the unannotated query from the user. The output of BLASTX run is an alignment file in a tsv format. The latter, is transformed into the protein list, as required input file for the DAVID and QuickGO web-services.

#### Assignment of Functional and Gene ontology annotation from DAVID

DAVID client application, retrieves the functional and gene ontology annotation for every single transcript in a query dataset. These python scripts take the protein’s list from the previous step and utilize DAVID database to obtain information in the form of ChartReport, ClusterReport, TableReport and SummaryReport. For a given query dataset, Python source code implemented within the Transcriptator pipeline runs with default parameters to obtain the enrichment statistics for each functional and GO term from DAVID database. ChartReport is an annotation-term-focused view, which lists annotation terms and their associated genes under study. It also provides the Fischer exact statistics calculated for each annotation term and information about the statistically enriched annotation terms in the query dataset. The ClusterReport displays the grouping of similar annotation terms along with their associated genes. The grouping algorithm is based on the hypothesis that alike annotations should have similar gene members. Trancriptator carries out functional annotation analysis in two separate modes with respect to the input data provided by the users. In case of fasta sequences, for the de-novo assembled transcripts for which no gene-related information is available, it carries out a comparative genomics approach, using BLASTX run for the given transcripts to obtain the closest protein hits from the UNIPROT/SWISSPROT database. The functional enrichment analysis is carried out for these protein hits, and by default, the specie which provides the maximum hits for BLASTX run is taken as background population. On the other hand, if the user submits known protein or gene ids from a reference organism to the Transcriptator pipeline, the DAVID built-in web service is default set to select the reference organism as back ground population to perform the enrichment analysis. The Transcriptator also provides an additional functionality to the users to provide their own list of proteins or gene ids, which could be used as background population to carry out the enrichment analysis for the given list of proteins. In this scenario, the user has to provide two distinct lists of protein ids. The functionality is currently provided only in those cases, where id’s datasets are used as input data for annotation purpose in Transcriptator.

#### Assignment and analysis of GO slim terms from QuickGO

Transcriptator employs BioServices module from Python package, which provides access to many bioinformatics web services and a framework to easily implement web service wrappers (based on WSDL/SOAP or REST protocol). BioServices (bioservices.quickgo.QuickGO) are used to investigate the GO-Slim in the query dataset. GO-Slim terms are the list of GO terms that have been selected from the full set of terms available from the gene ontology projects.

#### Processing of retrieved annotation

Both DAVID and QuickGO web services can produce large amount of results for the given query dataset. For the integration and summarization of retrieved results from web-services, Python and R codes in Transcriptator are implemented to parse the results in simpler format. Transcriptator produces easy to read tables for enrichment analysis of GO and Functional terms, clustering analysis on transcripts and annotation assignment for every single transcript. R scripts are specifically implemented in the pipeline, to generate an interactive chart for the distribution of functional and GO terms such as biological process, molecular function and cellular components associated with the query dataset of transcripts.

#### ncRNA prediction methodology

Transcriptomics analysis of an organisms not only provides the gene expression profiles, but also addresses the structural genomic informations for the organisms which does not have well annotated genomes. Previous research works have shown the differential expression of ncRNA in developmental and tissue specific condition and also their abnormal transcriptional rewiring in various diseases, including cancer [29–31]. At present, biologists are using high throughput sequencing of transcripts (RNA-seq) to detect the ncRNA. RNA-seq has been experimented on a number of model genomes and it has contributed to the identification of novel ncRNAs in these species. Somehow, in case of de-novo assembled transcriptome of non-model organism, for which well annotated reference genomes is not available, it is difficult to predict and annotate the ncRNAs. It is possible to determine non-coding RNAs which are differentially expressed and may or may not be part of substantial remodeling of transcriptional response. Non-coding RNAs, unlike messenger RNAs, do not code for protein products but instead perform unique functions by folding into higher order of structural conformations and have regulatory effects on gene expression at both pre and post trancriptional stage. To detect these ncRNAs, there are several methodologies and softwares, improvising evolutionary, statistical, machine learning methods were developed [12, 32–36]. We implemented an alternative pipeline to predict non coding RNAs. Using the code, the coding potential of each transcript is evaluated by a Support Vector Machine (SVM). The prediction of non-coding transcripts does not require homology information, it is generally supported by two ab initio models such as protein dependent SVM and protein independent SVM model [12, 35] for protein coding and non-coding transcripts respectively. This section of our pipeline takes input data as fasta sequences of unknown transcripts. It carries out the ncRNA prediction using built-in SVM model and generates several output files. The prediction results for the ncRNA, for a given input multi fasta sequence dataset is provided in the form of probability rather than p-value on hypothesis test. High probability for non coding prediction suggests a higher reliability of results. The results for the ncRNA prediction are generated in a tabular format, as well as dynamical graphs, for which the code is implemented in JAVA.

### Web Interface

**Fig 3:**
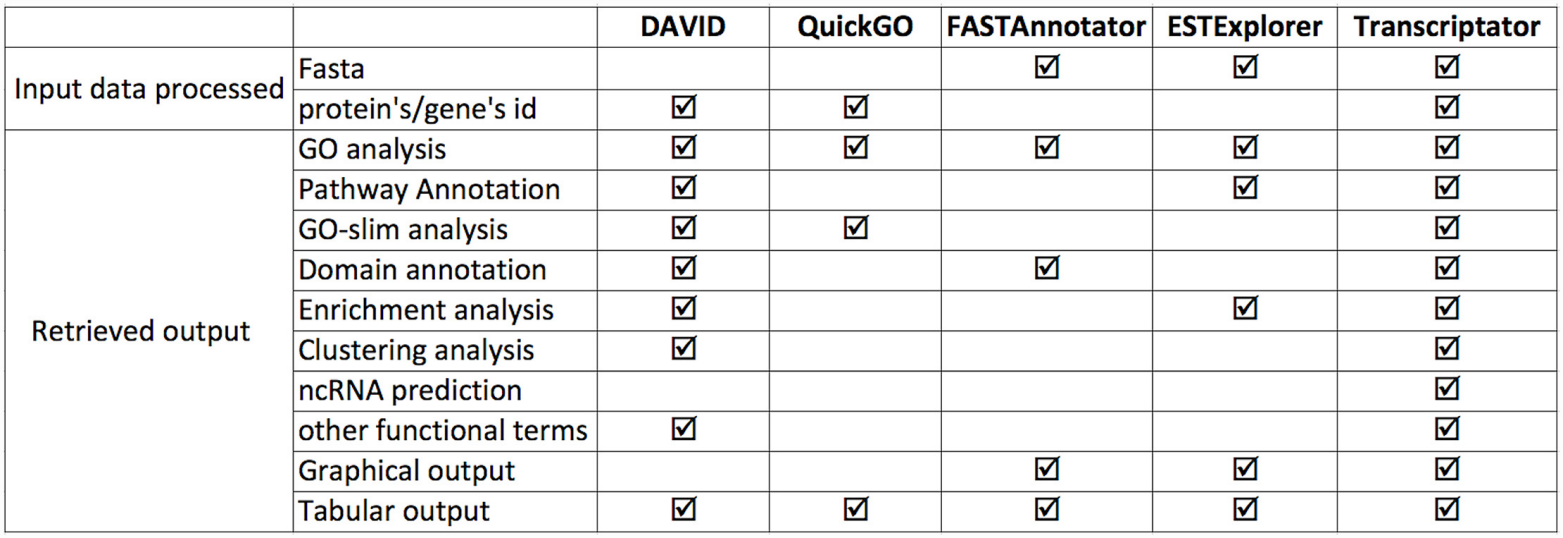
Transcriptator-pipeline-assessment.

Trancriptator web application is designed using ZK framework (http://www.zkoss.org/download/zk) and J2EE (Java 2 Platform Enterprise Edition, www.oracle.com/technetwork/java/javaee) technologies. The modular and distributed J2EE platform is employed to integrate technologies for the exchange of information between different applications, such as XML and Web Services. The implementation of the Graphical User Interface (GUI) is obtained using ZK framework, Ajax web application open-source, with XUL/XHTML (XML User Interface Language/Extensible HyperText Markup Language) built-in based components. JFASTA library v. 2.1.2 (http://jfasta.sourceforge.net/) is used to handle FASTA format files (.fa). BIOJAVA3-ws module (http://www.biojava.org/docs/api/org/biojava3/ws) of BIOJAVA v. 3.0.7 API is used to provide analytical and statistical routines, sequences manipulation such as BLAST alignment. Lastly, the Jython interpreter v. 2.5.3. (http://www.jython.org/) is used to integrate Python′s pipeline (Fig 3) code on Java′s platform.

## Results and Discussions

Transcriptator web application provides a user friendly interface to input differentially expressed unannotated transcripts or de-novo assembled reads from RNA-seq experiments in multi fasta file format as well as protein/gene ids from the reference organisms. To show the usefulness of our pipeline with respect to the other existing tools, we carried out a comparative functionality assessment, as shown in Fig 3. The built-in flexibity to input different data formats, the integrated functional annotation, and the non coding RNA prediction make Transcriptator pipeline a valuable software tool for bioinformatics analysis.

**Fig 4:**
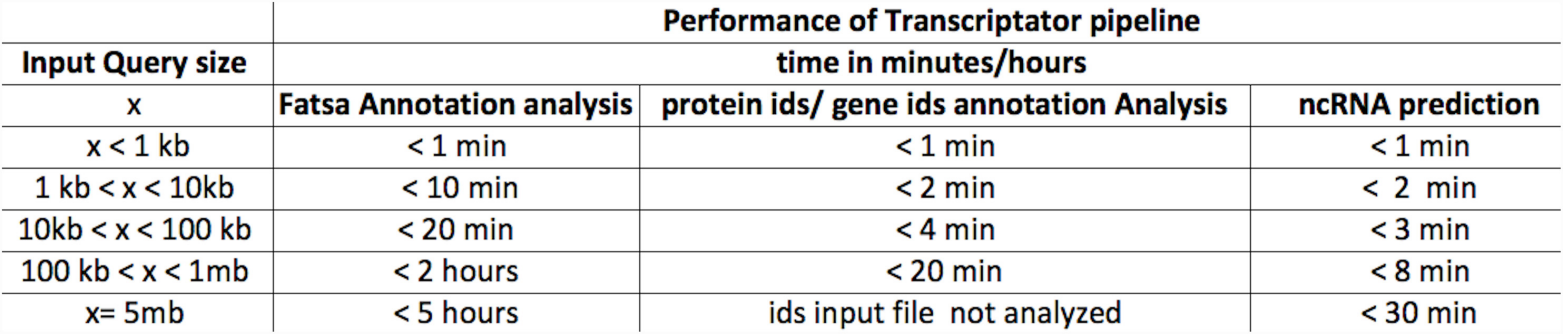
Performance of Transcriptator pipeline: it summarizes the computational efficiency of our pipeline, with respect to time, to generate the results for different analysis, for the input data of various sizes.

Since DAVID web service limits the analysis to three thousand id’s at a given time, our web server also allows the user to input up to three thousand transcripts sequences or id’s for annotation. To test the pipeline, we carried out functional annotation analysis of five hundred and fourty four transcripts as well as id’s dataset of more than four hundred genes. Both the tests run successfully and generated all the results through our pipeline (for the details see case study section). After a successful submission, a unique job ID is generated and provided as an identifier to start the annotation. All annotation results from DAVID and QuickGO are obtained through our server and the user can download them using the associated job ID. The results for single job id comprise of several tables and graphs. The results for functional annotation are divided into three sections. The first section contains a table with the list of the best hit proteins with e-value from the databases for the corresponding transcript. The second section comprises of a tables generated from the DAVID annotation analysis, such as: chartReport (for enrichment analysis); clusterReport (for clustering analysis); tableReport for functional and GO annotations for every single transcripts in the dataset; summaryReport with the summary of total annotations for the given query dataset. The third section comprises of the table enlisting the assignment of GO slim terms on the transcripts. The pipeline also produces charts related to the distribution of GO terms specifically related to three categories of biological processes, molecular function and cellular components, respectively, for the input query dataset. For each finished job, the related annotation results will be retained for one week on the Transcriptator server. User can access to this information by using the job id’s provided by the server. To test the computational efficiency of the different sections of Transcriptator pipeline, we carried out test runs for the different kind of analysis, for distinct input data of various sizes. The tabular results in Fig 4 show the execution time to generate the results for these tests.

### Case study 1

To demonstrate the utility of Transcriptator in biological studies, we have selected a sample dataset (five hundred and forty four unannotated transcripts) of *Hydra vulgaris* transcriptome, downloaded from European Nucleotide Archieve (ENA) database (http://www.ebi.ac.uk/ena/) which has recently been deposited at the ENA under the project number PRJEB445 and with accession numbers from HAAC01000001–HAAC01045269 [37]. These transcripts are specifically differentially expressed in response to cadmium treatment (unpublished data of specific differentially expressed transcripts for cadmium treatment). Cadmium is a toxic element. It accumulates in the organisms body and produces pathogenic changes. To study the harmful effects of cadmium accumulation in the body, previously researchers have studied the toxicity and chemical stress due to cadmium concentration in non model organism *Hydra* [38]. They have shown morphological, developmental and physical damage in *Hydra* due to the presence of high concentration of cadmium in the organism body. To undermine the molecular mechanism of cadmium poisoning in *Hydra*, we have investigated these cadmium specific differentially expressed transcripts through our pipeline Transcriptator. We obtained number of results for the given transcripts dataset. The results are divided into number of sections for the easy inference and analysis of the data.

#### Enrichment analysis

**Fig 5:**
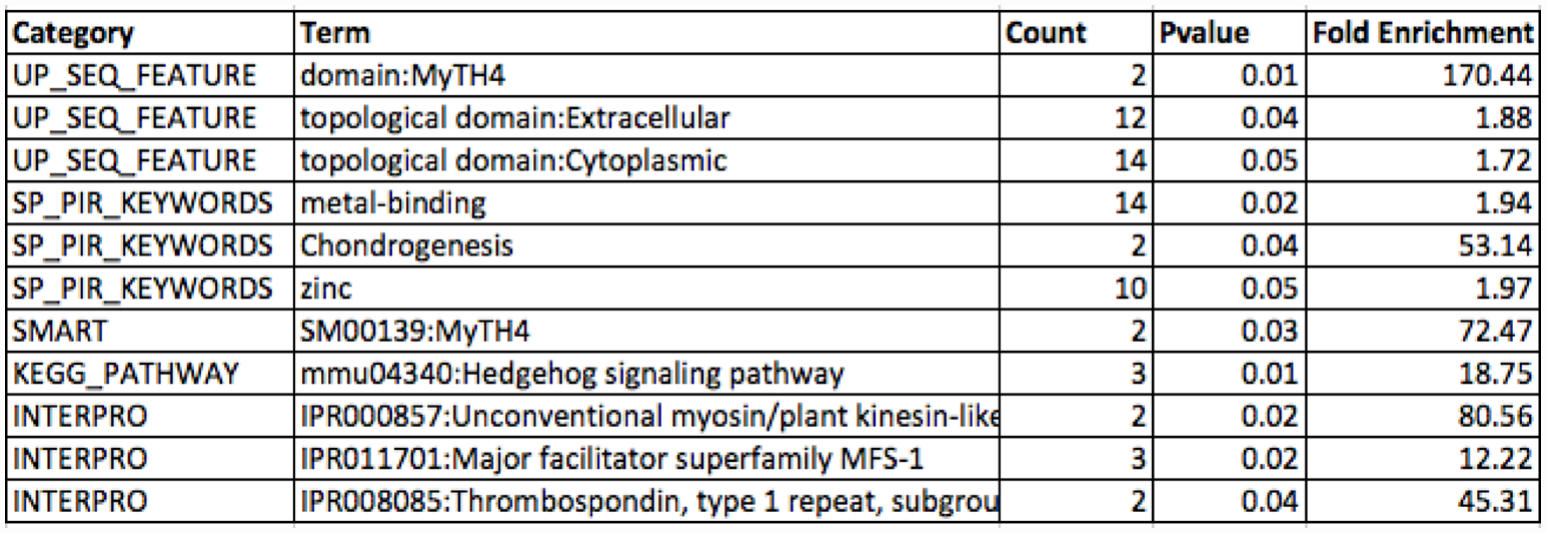
Enrichments analysis of functional annotation terms which are significantly distributed among the test data set of *Hydra vulgaris* transcripts.

We first calculated the enrichment of functional annotation terms. The statistical p-value is further corrected by benjamini and bonferroni multiple testing methods and the cutoff is taken as ⩽ 0.05. We obtained the various categories of functional annotation terms which are enriched within the list of five hundred and forty four transcripts. In Fig 5, we are showing the top enriched terms with the best p-values. These terms are related to Uniprot sequence feature, SMART and INTERPRO domains and KEGG pathway. It is obvious from the enrichment results, UP-SEQ FEATURE:MyTH4 domain is highly enriched with a fold change around 170. The other significant functional annotation terms are SP-PIR keyword: metal binding (fold enrichment 1.94), Hedgehog signaling pathway (fold enrichment 18.75) and INTERPRO domains such as unconventional myosin domain (fold enrichment 80.56) and major facilitator superfamily MFS1 domain (fold enrichment 12.22).

The MyTH4 domain is present in microtubule-based kinesin motors and actin-based myosin motors proteins, which generate movements required for intracellular trafficking, cell division, and muscle contraction. There is an evidence that the MyTH4 domain of Myosin-X (Myo10) binds to microtubules and thus could provide a link between an actin-based motor protein and the microtubule cytoskeleton [39]. The microtubule cytoskeleton is responsible for the structure of cell and we observed the differential regulation in these proteins during the morphological, developmental and physical damage in *Hydra* in response to cadmium poisoning. The role of myosin protein is also implicated in the elongation of filopodia, which function as tentacles that explore and interact with cell surroundings to determine the direction of cell movement and to establish cell adhesion [40]. It also plays an important role in regeneration process of *Hydra* and its embryogenesis [41]. It was found that heavy metals such as copper, cadmium and zinc were accumulated in the tissues of *Hydra* during direct exposure to the metals in water and also indirectly through feeding on contaminated prey [42]. With the previous understanding of heavy metal accumulation in the Hydra tissue, we also obtained “metal binding” terms as enriched. We also retrieved the Hedgehog signaling pathway as enriched KEGG pathway in our transcripts dataset. It is a signaling pathway that transmits information to embryonic cells required for proper development. The Hedgehog signaling pathway is one of the key regulators of animal development and is present in all bilaterians. Recent studies, define the role of Hedgehog signaling in regulating adult stem cells, involved in maintenance and regeneration of adult tissues [43]. Hedgehog (Hh) family of secreted signaling proteins plays a crucial role in development and morphogenesis of a variety of tissues and organs in *Hydra vulgaris*.

#### Clustering analysis

**Fig 6:**
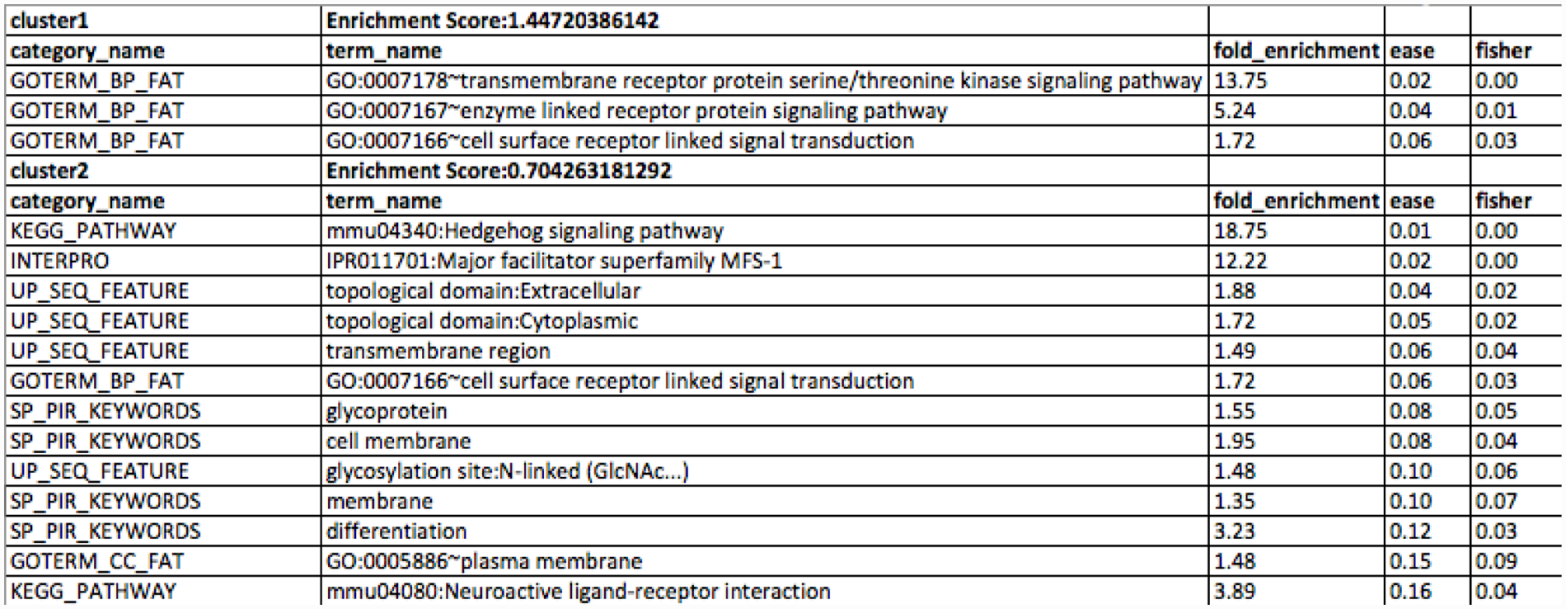
Clustering of annotation terms shared by the common set of deregulated genes.

We determine the common functionalities within the deregulated genes, in response to cadmium treatment in *Hydra vulgaris*. These results help users, to understand the over all behavior of transcriptomic regulation. It helps in the classification of the differentially regulated genes, on the basis of common functional repertoire. For example in Fig 6, we have shown top two clusters, representing two broader categories of functionalities such as protein signal transduction and membrane transport as significant. These functional terms are enriched within the common set of genes. The cut off value for clustering is taken as enrichment score greater than 0.70. This analysis put a picture of transcriptomic regulation under a given experimental condition on a broader spectrum.

#### Annotation reports

Transcriptator does not only provide an annotation details for the Gene Ontology (GO) terms, but also try to cover several other realms of functionalities such as pathways, domains and also GO-Slim terms. All these informations help users to make their biological understanding much better. For example, in case of the given query dataset, the GO terms annotation table (see S1 Table) shows all the gene ontology terms belonging to biological processes, molecular functions and cellular components, specifically associated to each transcript represented by their most closest homologous protein. In our results, we also provide parsed blast result to the users, so they can easily identify the closest homologous protein for each given transcript. In pathway annotation table (see S3 Table), KEGG and Panther annotations information are provided to the user. Similarly, domain (see S2 Table) report shows the annotations details for the INTERPRO, SMART, PIR domains with respect to each closest protein associated to the transcript. Trancriptator pipeline also provide another table for GO-Slim terms (see S4 Table), whereas specific GO terms are merged into broader categories to remove the redundant information.

#### Distribution Plots

**Fig 7:**
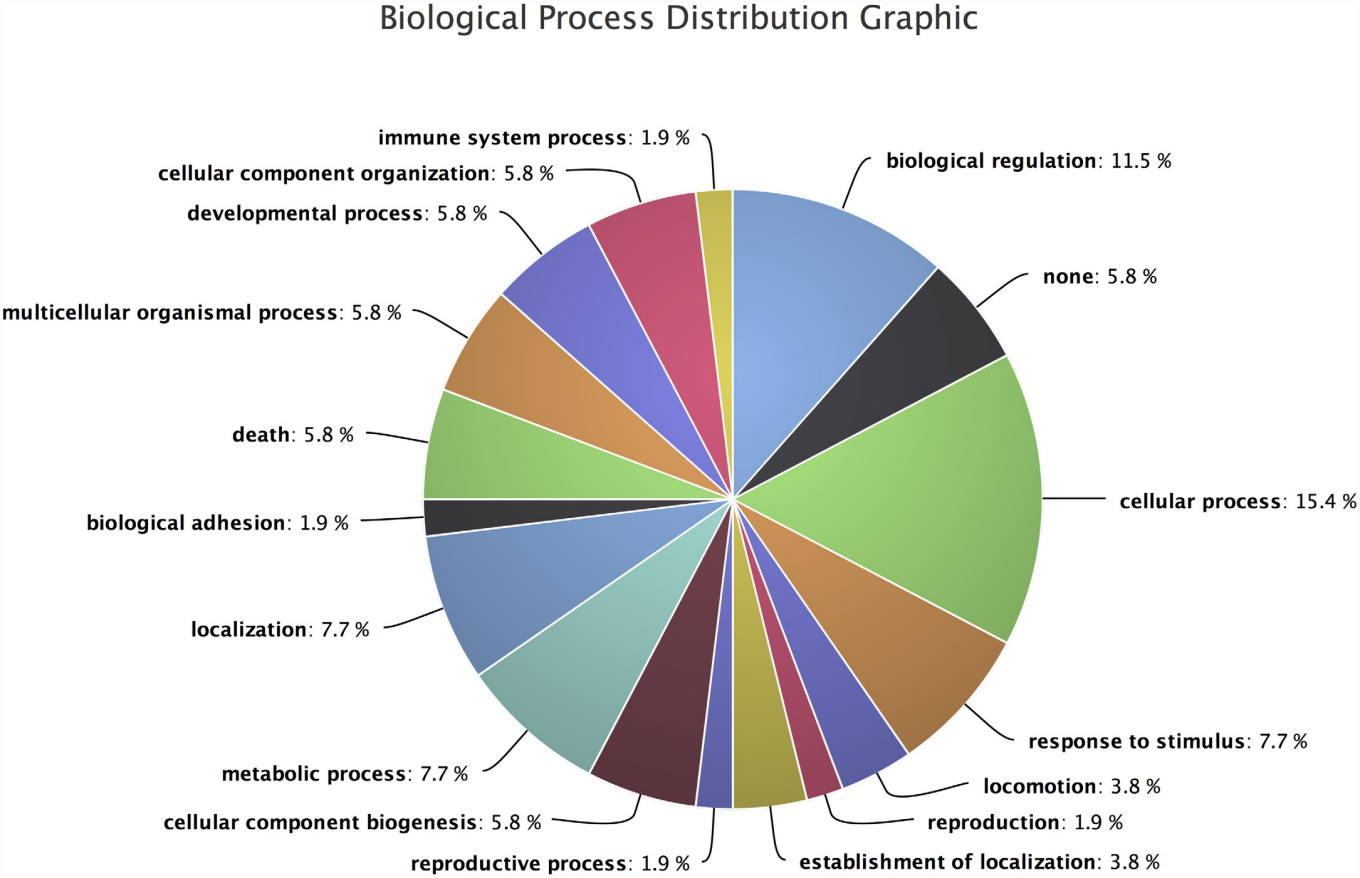
Biological processes distribution for the *Hydra* dataset. It shows the significant biological activities, in which these transcripts are involved. For example, the biological regulation, cellular process, stimulus response activities and developmental process are enriched within these transcripts.

Along with the tabular reports, Transcriptator pipeline produces a series of dynamic distribution plots. Our pipeline collects the annotation data from web-services, parses it and visualizes the distribution of GO terms, protein domains, Pathways, and other functionalities within the given set of transcripts. The Biological Process (BP) distribution plot (Fig 7) shows the frequency distribution of prominent biological processes. BP terms such as: biological regulation (14.2%), cellular process (19.2%), stimulus response activities(5.7%) and developmental process(6.1%) are enriched within the given set of the transcripts. It is important to note that immune system (1.2%), growth (0.4%), death (1.9%) and biological adhesion (1.0%) are some other biological processes which are important though not prominently distributed with in the given cadmium treated differentially expressed transcripts dataset.

**Fig 8:**
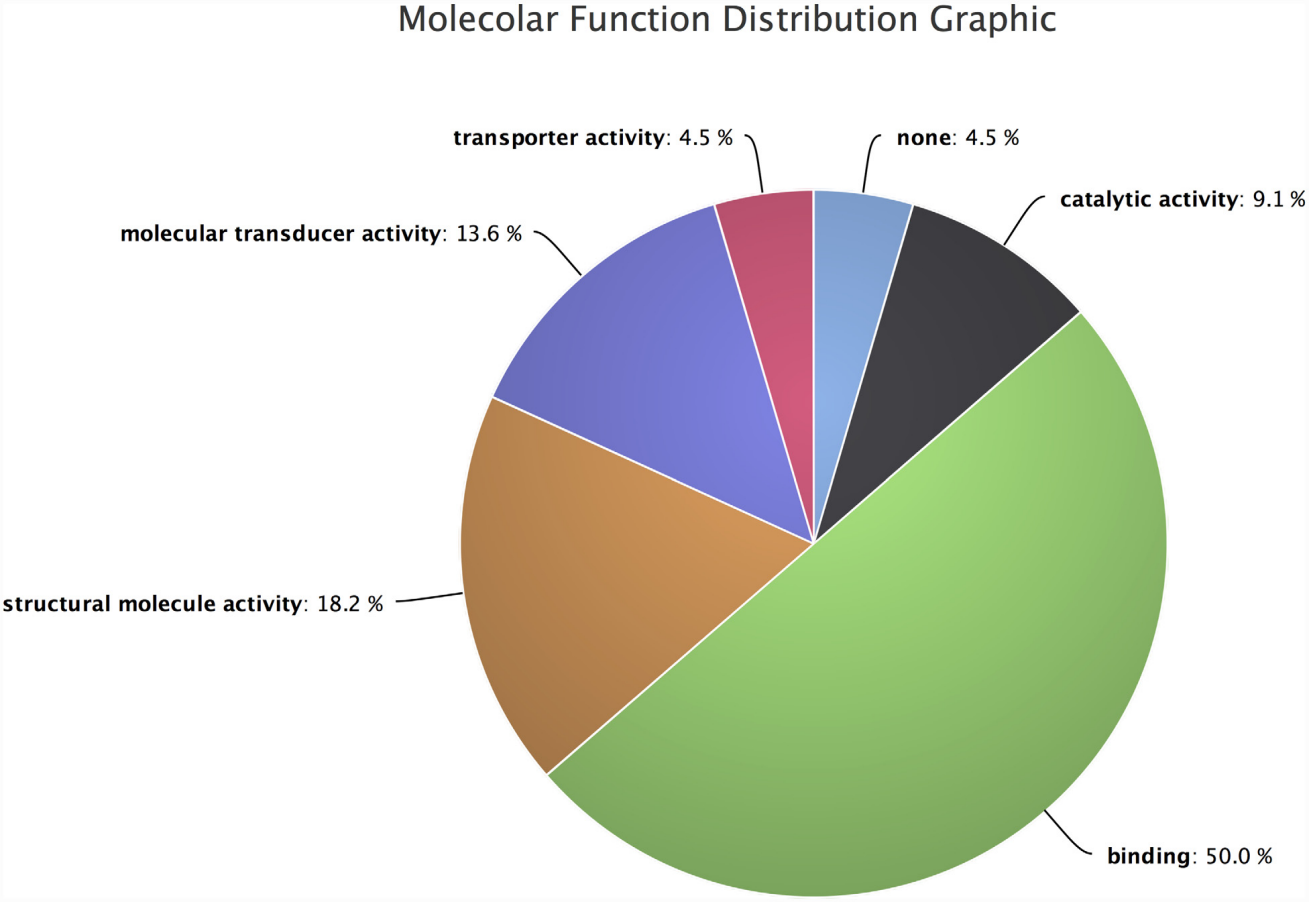
Molecular function distribution for the *Hydra* dataset. It shows the significant molecular function activities, in which these transcripts are involved. For example binding, molecular transducer activity, transcription regulator activity and catalytic activities are enriched within these transcripts.

Molecular functions distribution plots (Fig 8) suggests 42.6% of sample dataset of *Hydra* transcripts involved in binding function. It shows transcription regulator activity (4.6%), transporter activity (6.6%), catalytic activity (16.7%) and molecular transducer activities (11.5%), which are also enriched in these transcripts dataset of *Hydra* in response to the cadmium toxicity.

**Fig 9:**
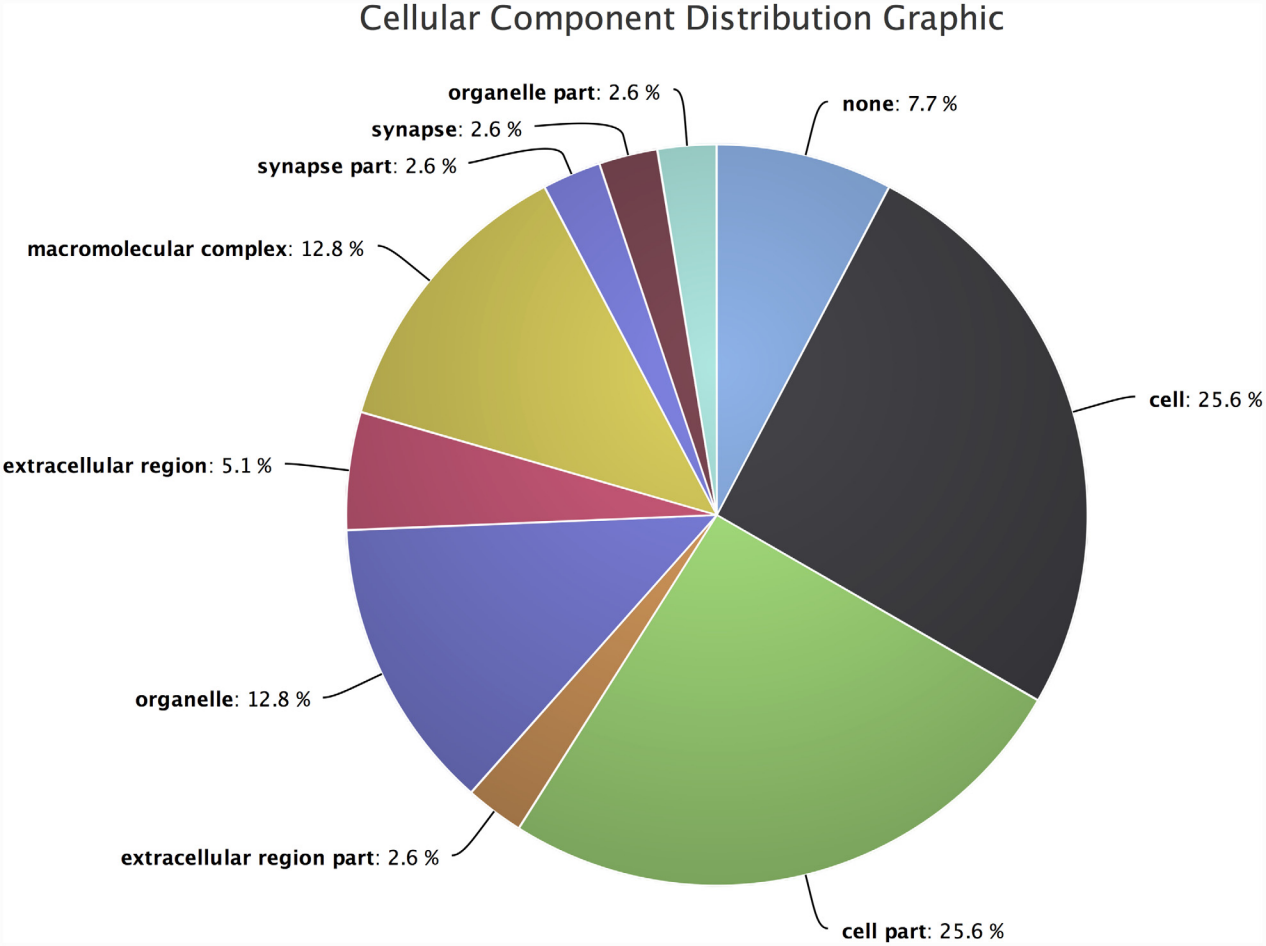
Cellular components distribution for the *hydra* dataset. It shows, most of the differentially expressed transcripts from the query dataset are associated with cell organization. A small number of transcripts are also involved with structural composition of synapse, macromolecular complex, membrane and cellular organelle, but are not statistically significant.

Distribution of cellular components (Fig 9), suggests the role of these transcripts in cellular composition. It shows the percentage distribution of the cellular components such as synapse, macromolecular complex region, organelles and membrane enclosed regions. Unfortunately, these cellular components terms are not enriched in our query dataset.

**Fig 10:**
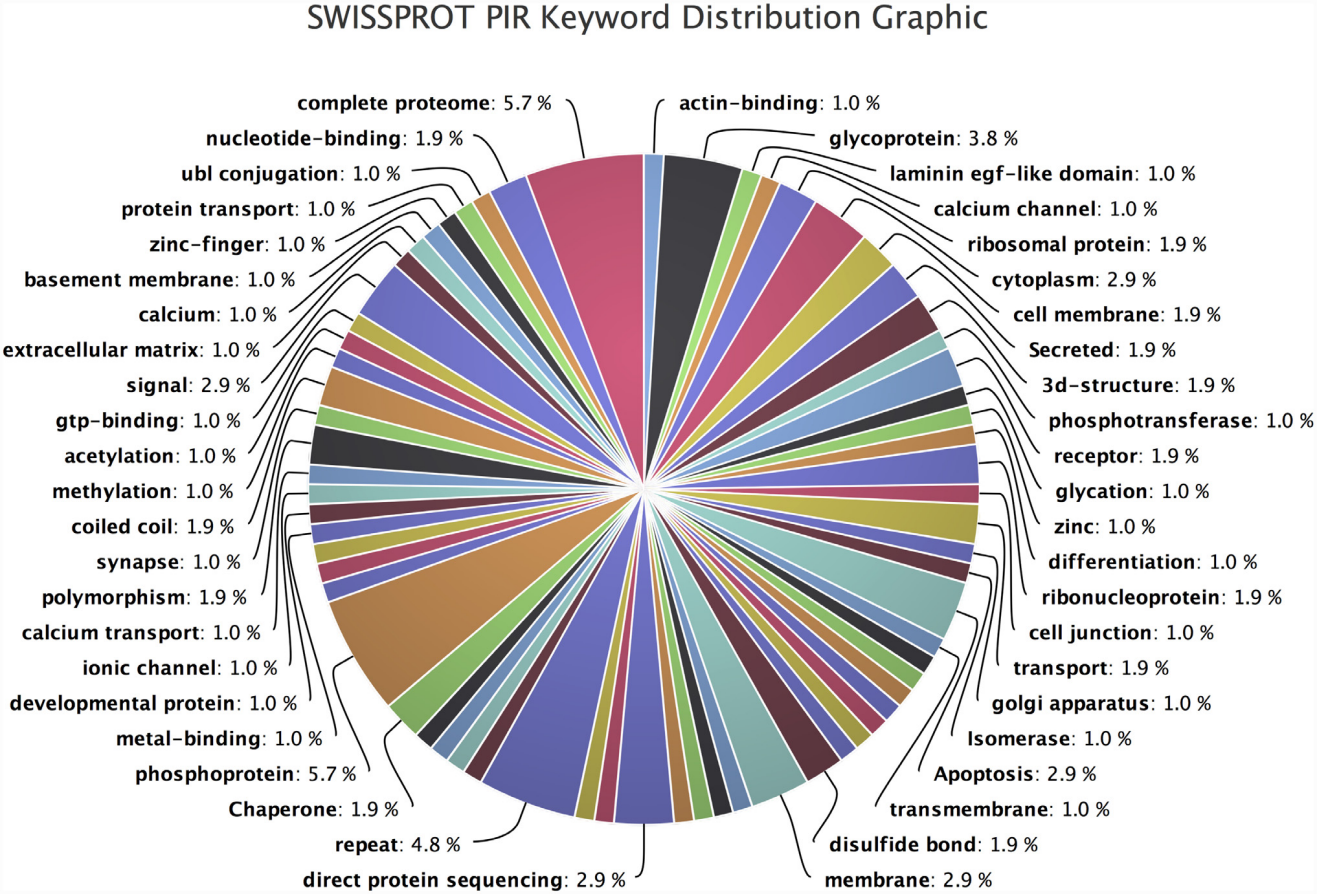
SwissProt-PIR keywords distribution: Graphical representation of all the SP-PIR keywords.

Annotation results for the Biocarta pathways, Panther pathways, KEGG pathways, proteins domains like InterPro, PFAM, SMART and SP-PIR-keywords are also obtained. These results are provided in the form of distribution plots, as well as annotation tables. For example, SP-PIR-keyword distribution plot (Fig 10) shows a large number of keywords, which are associated to the query dataset. It includes terms like transcription, transducer, alternative splicing, differentiation, DNA binding, developmental proteins, g-protein coupled receptor, nucleotide binding, signal and ion transport etc. All these terms, associated to the differentially expressed transcripts in the dataset, indicates the possible role of cadmium toxicity on differentiation, reproduction, developmental and signal transduction processes in *Hydra vulgaris*.

### Case study 2

To demonstrate the reliability of Transcriptator in carrying out enrichment analysis of GO terms, we obtain already published dataset (GEO Series accession number GSE7535) of cadmium-responsive up-regulated genes from *Caenorhabditis elegans*. These genes have been mapped to biological processes and molecular functions following 24 hours cadmium exposures [44]. We carried out molecular function enrichment analysis for the given set of gene’s dataset and obtained electron carrier activity (GO:0009055), heme binding (GO:0020037), cation binding (GO:0043169) as significant enriched molecular function terms. Our results are in concordance with the earlier published research. The enrichment analysis table is provided as supporting evidence (see S5 Table)).

### Case study 3: ncRNA Prediction

**Fig 11:**
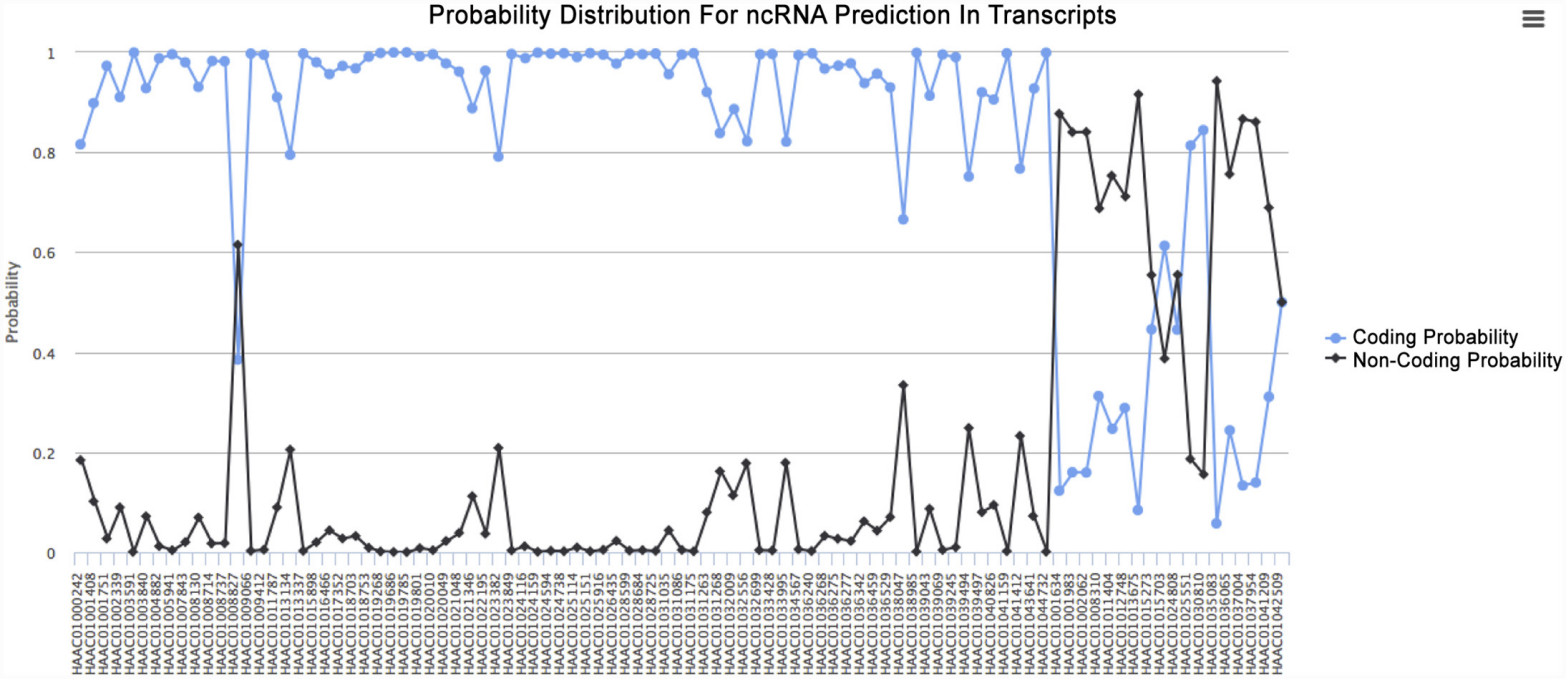
ncRNA prediction: graphical chart for the prediction of coding and non-coding transcripts. Each transcript is represented by two probability graphs for coding (**blue**) and non-coding (**black**) respectively.

To demonstrate the utility of ncRNA prediction pipeline in Transcriptator, a sample dataset of de-novo assembled *Hydra* Transcripts from European Nucleotide Archive (ENA) was selected. This dataset contains one hundred and one differentially expressed transcripts in response to cadmium treatment for eight and twenty four hour time period (unpublished experiment). To predict the coding and non-coding probability of these differentially expressed transcripts, we carried out ncRNA prediction analysis through our Transcriptator software pipeline. It helps us in understanding the behavior of transcriptional response of *Hydra vulgaris* transcripts under cadmium treatment. For one hundred and one transcripts, transcriptator pipeline for ncRNA prediction provides result for 93 transcripts (around 93%), while a log file is generated for the erroneous transcripts. The results from this ncRNA prediction functionality provides tables for the probability score for ncRNA prediction (see S6 Table), and dynamical graph (Fig 11) showing the probability for coding and non-coding characteristics of the given transcripts. It also provides separate fasta files for coding and non-coding transcripts for further usage in the downstream analysis.

In this study, we have developed an integrated web application to carry out functional and GO annotation analysis for the given coding transcripts but also provided an opportunity to the biologist to address transcripts which are non coding in nature. It allows users to choose two distinct types of web services for annotation purposes, as well as different BLAST databases for BLAST run. In the light of NGS sequencing technologies, when there is a paradigm shift in the understanding of transcriptional regulation due to the presence of non coding element expression with in the transcriptomic data. It becomes vital to understand and acknowledge the importance of non-coding transcripts differential expression. Therefore, we have also introduced a parallel processing of non-coding RNA prediction, by using a well known Support Vector based Machine learning program PORTRAIT in our existing pipeline. All these options helps the users, to optimize their results according to their needs. It provides the enrichment score for the functional terms, and reports each and every annotation present in the given dataset in the form of tables and interactive charts. It also provides the dynamic chart for coding and non-coding probabilities for the given transcripts dataset. The results are generated in the form of fasta formated files, Charts and tabular reports, so that, the resultant files could be used for down-stream analysis. We propose that our web application does not have only technical aspects, but it can be very helpful to the researchers, to elaborate and define the biological meaning of the transcriptomic data. In future, we will work on the addition of more modular functionalities and options in the pipeline, for both BLAST searches and annotation analysis for coding as well as non-coding transcripts.

## Supporting Information

S1 Table Case study 1: GO-terms.xlsx. This table contains the available GO annotations for each transcript given in *Hydra vulgaris* transcripts dataset.
(XLSX)

S2 Table Case study 1: Domains.xlsx. This table contains the available domain annotations for each transcript given in *Hydra vulgaris* transcripts dataset.
(XLSX)

S3 Table Case study 1: Pathways.xlsx. This table contains the available pathway annotations for each transcript given in *Hydra vulgaris* transcripts dataset.
(XLSX)

S4 Table Case study 1: GO-SLIM.xlsx. This table contains the available GO-slim annotations for each transcript given in *Hydra vulgaris* transcripts dataset.
(XLSX)

S5 Table Case study 2: MF-enrichment.pdf. This table shows the significant molecular function associated with *Caenorhabditis elegans* genes dataset which are up-regulated in response to 24 hour cadmium exposure.
(PDF)

S6 Table Case study 3: ncRNA-prediction-scores.pdf. This table contains the probability score for coding and non-coding characterstics for each transcript given in *Hydra vulgaris* transcripts dataset.
(PDF)

## References

[1] Steijger T, Abril JF, Engström PG, Kokocinski F, The RGASP Consortium, Hubbard TJ, et al Assessment of transcript reconstruction methods for RNA-seq. Nat Methods. 2013;10:1177–1184. 10.1038/nmeth.2714 24185837 PMC3851240

[2] Huang da W, Sherman BT, Lempicki RA. Systematic and integrative analysis of large gene lists using DAVID bioinformatics resources. Nature Protocols. 2009;4:44–57. 10.1038/nprot.2008.211 19131956

[3] Binns D, Dimmer E, Huntley R, Barrell D, O’Donovan C, Apweiler R. QuickGO: web-based tool for Gene Ontology searching. Bioinformatics. 2009;25(22):3045–3046. 10.1093/bioinformatics/btp536 19744993 PMC2773257

[4] Nagaraj SH, Deshpande N, Gasser RB, Ranganathan S. ESTExplorer: expressed sequence tag (EST) assembly and annotation platform. Nucleic Acids Res. 2007;35 (Web Server issue):143–147. 10.1093/nar/gkm378 17545197 PMC1933243

[5] Chen TW, Gan RC, Wu TH, Huang PJ, Lee CY, Chen YY, et al FastAnnotator- an efficient transcript annotation web tool. BMC Genomics. 2012 12;13(7):S9. 10.1186/1471-2164-13-S7-S9 PMC3521244 23281853

[6] Wang X, Cairns MJ. Gene set enrichment analysis of RNA-seq data: integrating differential expression and splicing. BMC Bioinformatics. 2013;14(5):S16 10.1186/1471-2105-14-S5-S16 23734663 PMC3622641

[7] Huang da W, Sherman BT, Lempicki RA. Bioinformatics enrichment tools: toward the comprehensive functional analysis of large gene lists. Nucleic Acids Res. 2009;37(1):1–13. 10.1093/nar/gkn923 19033363 PMC2615629

[8] Altschul SF, Gish W, Miller W, Myers EW, Lipman DJ. Basic local alignment search tool. J Mol Biol. 1990;215:403–410. 10.1016/S0022-2836(05)80360-2 2231712

[9] Apweiler R, Bairoch A, Wu CH, Barker WC, Boeckmann B, Ferro S, et al UniProt: The Universal Protein knowledgebase. Nucleic Acids Res. 2004;32:115–119. 10.1093/nar/gkh131 14681372 PMC308865

[10] Cokelaer T, Pultz D, Harder LM, Serra-Musach J, Saez-Rodriguez J. BioServices: common Python package to access biological Web Services programmatically. Bioinformatics. 2013;29:3241–3242. 24064416 10.1093/bioinformatics/btt547 PMC3842755

[11] Jiao X, Sherman BT, Huang da W, Stephens R, Baseler MW, Lane HC, et al David-ws: stateful web service to facilitate gene/protein list analysis. Bioinformatics. 2012;28(13):1805–1806. 10.1093/bioinformatics/bts251 22543366 PMC3381967

[12] Arrial RT, Togawa RC, Brigido MM. Screening non-coding RNAs in transcriptomes from neglected species using PORTRAIT: case study of the pathogenic fungus Paracoccidioides brasiliensis. BMC Bioinformatics. 2009;10:239 10.1186/1471-2105-10-239 19653905 PMC2731755

[13] Cortes C, Vapnik V. Support-Vector Networks. Machine Learning. 1995;20:273–297. 10.1023/A:1022627411411

[14] Kanehisa M, Goto S. Kegg: Encyclopedia of Genes and Genomes. Nucleic Acids Res. 2000;28(1):27–30. 10.1093/nar/28.1.27 10592173 PMC102409

[15] Thomas PD, Campbell MJ, Kejariwal A, Mi H, Karlak B, Daverman R, et al PANTHER: A Library of Protein Families and Subfamilies Indexed by Function. Genome Res. 2003;13:2129–2141. 10.1101/gr.772403 12952881 PMC403709

[16] Letunic I, Doerks T, Bork P. Smart: recent updates developments and status in 2015. Nucleic Acids Res. 2014;43:257–260. 10.1093/nar/gku949 PMC4384020 25300481

[17] Sigrist CJ, de Castro E, Cerutti L, Cuche BA, Hulo N, Bridge A, et al New and continuing developments at PROSITE. Nucleic Acids Res. 2012;41:344–347. 10.1093/nar/gks1067 PMC3531220 23161676

[18] Servant F, Bru C, Carrère S, Courcelle E, Gouzy J, Peyruc D, et al ProDom: clustering of homologous domains. Briefings in Bioinformatics. 2002;3(3):246–251. 10.1093/bib/3.3.246 12230033

[19] Finn RD, Bateman A, Clements J, Coggill P, Eberhardt RY, Eddy SR, et al The Pfam protein families database. Nucleic Acids Res Database Issue. 2014;42:222–230. 10.1093/nar/gkt1223 PMC3965110 24288371

[20] Mitchell A, Chang HY, Daugherty L, Fraser M, Hunter S, Lopez R, et al The InterPro protein families database: the classification resource after 15 years. Nucleic Acids Res Database issue. 2014;43:213–221. 10.1093/nar/gku1243 PMC4383996 25428371

[21] Licata L, Briganti L, Peluso D, Perfetto L, Iannuccelli M, Galeota E, et al MINT, the molecular interaction database:update. Nucleic Acids Res Database issue. 2012;40:857–861. 10.1093/nar/gkr930 PMC3244991 22096227

[22] Bader GD, Betel D, Hogue CWE. Bind: Biomolecular Interaction Network Database. Nucleic Acids Res. 2003;31(1):248–250. 10.1093/nar/gkg056 12519993 PMC165503

[23] Gibb EA, Brown CJ, Lam WI. The functional role of long non-coding RNA in human carcinomas. Molecular Cancer. 2011;10:38 10.1186/1476-4598-10-38 21489289 PMC3098824

[24] Rosa A, Brivanlou AH. Regulatory Non-Coding RNAs in Pluripotent Stem Cells. Int J Mol Sci. 2013;14(7):14346–14373. 10.3390/ijms140714346 23852015 PMC3742248

[25] Villegas VE, Zaphiropoulos PG. Neighboring Gene Regulation by Antisense Long Non-Coding RNAs. Int J Mol Sci. 2015;16(2):3251–3266. 10.3390/ijms16023251 25654223 PMC4346893

[26] Wang J, Zhang J, Zheng H, Li J, Liu D, Li H, et al Mouse transcriptome: neutral evolution of’non-coding’ complementary DNAs. Nature. 2004;431(7010):1p. 10.1038/nature03016 15495343

[27] Mercer TR, Dinger ME, Mattick JS. Long non-coding RNAs: insights into functions. Nature Rev Genet. 2009;10(3):155–159. 10.1038/nrg2521 19188922

[28] Bakel HV, Nislow C, Hughes TR. Most “dark matter” transcripts are associated with known genes. PLoS Biol. 2010;8:5 10.1371/journal.pbio.1000371 PMC2872640 20502517

[29] Taft RJ, Pang KC, Mattick JS. Non-coding RNAs: regulators of disease. J Pathol. 2010;220(2):126–139. 19882673 10.1002/path.2638

[30] Maruyama R, Shipitsin M, Choudhury S, Wu Z, Protopopov A, Yao J, et al Breast Cancer Special Feature: Altered antisense-to-sense transcript ratios in breast cancer. Proc Natl Acad Sci. 2012;109(8):2820–2824. 21098291 10.1073/pnas.1010559107 PMC3286925

[31] Wu SC, Kallin EM, Zhang Y. Role of H3K27 methylation in the regulation of lncRNA expression. Cell Res. 2010;20(10):1109–1116. 10.1038/cr.2010.114 20680032 PMC2949548

[32] Toffano-Nioche C, Luo Y, Kuchly C, Wallon C, Steinbach D, Zytnicki M, et al Detection of non-coding RNA in bacteria and archaea using the DETR′PROK Galaxy pipeline. Methods. 2013;63:60–65. 10.1016/j.ymeth.2013.06.003 23806640

[33] Liu J, Gough J, Rost B. Distinguishing protein-coding from non-coding RNAs through support vector machines. PLoS Genet. 2006;2(4). 10.1371/journal.pgen.0020029 PMC1449884 16683024

[34] Kong L, Zhang Y, Gao G. CPC: assess protein-coding potential of transcripts using sequence features and support vector machine. Nucleic Acids Res. 2007;35:345–349. 10.1093/nar/gkm391 PMC1933232 17631615

[35] Chang CC, Lin CJ. LibSVM: a library for support vector machines. ACM transactions on intelligent systems and technology. 2011;2 10.1145/1961189.1961199 Available from: http://www.csie.ntu.edu.tw/~cjlin/libsvm.

[36] Shimizu K, Adachi J, Muraoka Y. Angle: a sequencing errors resistant program for predicting protein coding regions in unfinished cDNA. J Bioinform Comp Biol. 2006;4(3):649–664. 10.1142/S0219720006002260 16960968

[37] Wenger Y, Galliot B. RNAseq versus genome-predicted transcriptomes: a large population of novel transcripts identified in an Illumina-454 Hydra transcriptome. BMC Genomics. 2013;14(204). 10.1186/1471-2164-14-204 23530871 PMC3764976

[38] Karntanut W, Pascoe D. The toxicity of copper, cadmium and zinc to four different Hydra (Cnidaria: Hydrozoa). Chemosphere. 2002;47(10):1059–1064. 10.1016/S0045-6535(02)00050-4 12137038

[39] Weber KL, Sokac AM, Berg JS, Cheney RE, Bement WM. A microtubule-binding myosin required for nuclear anchoring and spindle assembly. Nature. 2004;431(7006):325–329. 10.1038/nature02834 15372037

[40] Sousa AD, Cheney RE. Myosin-X: molecular motor at the cell’s fingertips. Trends in Cell Biology. 2005;15(10):533–539. 10.1016/j.tcb.2005.08.006 16140532

[41] Martin VJ, Littlefield CL, Archer WE, Bode HR. Embryogenesis in Hydra. Biol Bull. 1997;192(3):345–363. 10.2307/1542745 9212444

[42] Karntanut W, Pascoe D. A comparison of metal accumulation by the cnidarian Hydra vulgaris directly from water or through contaminated prey and effects upon reproduction and regeneration. Songklanakarin J Sci Technol. 2007;29(3):869–880.

[43] Ingham PW, Nakano Y, Seger C. Mechanisms and functions of Hedgehog signalling across the metazoa. Nat Rev Genet. 2011;12(6):393–406. 10.1038/nrg2984 21502959

[44] Cui Y, McBride SJ, Boyd WA, Alper S, Freedman JH. Toxicogenomic analysis of Caenorhabditis elegans reveals novel genes and pathways involved in the resistance to cadmium toxicity. Genome Biol. 2007;8(6):122 10.1186/gb-2007-8-6-r122 PMC2394766 17592649

