# Supplementary material for: Transcriptator: An Automated Computational Pipeline to Annotate Assembled Reads and Identify Non Coding RNA
Source: PLoS One. 2015 Nov 18;10(11):e0140268. doi: 10.1371/journal.pone.0140268 (PMC4651556; doi:10.1371/journal.pone.0140268)
Supplement: S5 Table — This table shows the significant molecular function associated with Caenorhabditis elegans genes dataset which are up-regulated in response to 24 hour cadmium exposure. (PDF) [file pone.0140268.s005.pdf]

Molecular Functional enrichment table for case study 2 dataset

term_name (Molecular Function)	fold_enrichment	ease	fisher	Bonferroni	Benjamini	raw_FDR	adjusted_FDR+
GO:0009055~electron carrier activity	7.27	0.00	0.00	0.00	0.00	0.00	0.00
GO:0020037~heme binding	8.95	0.00	0.00	0.00	0.00	0.00	0.00
GO:0046906~tetrapyrrole binding	8.83	0.00	0.00	0.00	0.00	0.00	0.00
GO:0005506~iron ion binding	5.33	0.00	0.00	0.00	0.00	0.00	0.00
GO:0046914~transition metal ion binding	1.40	0.07	0.05	1.00	0.42	14.14	56.91
GO:0043169~cation binding	1.27	0.13	0.10	1.00	0.58	24.15	78.27
GO:0043167~ion binding	1.27	0.13	0.10	1.00	0.56	24.76	79.21
GO:0046872~metal ion binding	1.25	0.16	0.12	1.00	0.59	28.99	84.90
GO:0008270~zinc ion binding	0.48	1.00	0.99	1.00	1.00	100.00	100.00
GO:0030246~carbohydrate binding	2.82	0.10	0.03	1.00	0.49	18.24	67.11
GO:0070011~peptidase activity, acting on L-amino acid peptides	2.34	0.03	0.01	0.96	0.41	6.56	31.26
GO:0070001~aspartic-type peptidase activity	9.36	0.04	0.00	0.98	0.41	7.64	35.51
GO:0004190~aspartic-type endopeptidase activity	9.36	0.04	0.00	0.98	0.41	7.64	35.51
GO:0008233~peptidase activity	2.17	0.05	0.02	0.99	0.41	9.60	42.72
GO:0008236~serine-type peptidase activity	3.94	0.17	0.04	1.00	0.60	31.24	87.36
GO:0017171~serine hydrolase activity	3.94	0.17	0.04	1.00	0.60	31.24	87.36
GO:0004175~endopeptidase activity	1.94	0.25	0.11	1.00	0.73	43.07	95.54
GO:0043492~ATPase activity, coupled to movement of substances	5.05	0.04	0.01	0.98	0.39	8.27	37.93
GO:0042626~ATPase activity, coupled to transmembrane movement of substances	5.05	0.04	0.01	0.98	0.39	8.27	37.93
GO:0015405~P-P-bond-hydrolysis-driven transmembrane transporter activity	4.70	0.05	0.01	0.99	0.38	9.87	43.68

Table: It shows the significant molecular function associated with Caenorhabditis elegans genes which are up-regulated in response to 24 hour cadmium exposure.
