# Supplementary material for: Transcriptator: An Automated Computational Pipeline to Annotate Assembled Reads and Identify Non Coding RNA
Source: PLoS One. 2015 Nov 18;10(11):e0140268. doi: 10.1371/journal.pone.0140268 (PMC4651556; doi:10.1371/journal.pone.0140268)
Supplement: S6 Table — This table contains the probability score for coding and non-coding characterstics for each transcript given in Hydra vulgaris transcripts dataset. (PDF) [file pone.0140268.s006.pdf]

Transcripts ID	Coding Probability	Non-coding Probability
HAAC01000242	0.815815	0.184185
HAAC01001408	0.898292	0.101708
HAAC01001751	0.972827	0.0271729
HAAC01002339	0.910365	0.0896347
HAAC01003591	0.999399	0.000601017
HAAC01003840	0.928155	0.0718451
HAAC01004882	0.987981	0.0120191
HAAC01005941	0.996084	0.00391645
HAAC01007843	0.979831	0.0201691
HAAC01008130	0.930546	0.0694537
HAAC01008714	0.982529	0.0174706
HAAC01008737	0.981958	0.0180422
HAAC01008827	0.385104	0.614896
HAAC01009066	0.997279	0.00272097
HAAC01009412	0.994981	0.00501884
HAAC01011787	0.910229	0.0897715
HAAC01013134	0.794914	0.205086
HAAC01013337	0.997354	0.00264642
HAAC01015898	0.980028	0.0199718
HAAC01016466	0.956343	0.0436571
HAAC01017352	0.972632	0.0273675
HAAC01018703	0.96766	0.03234
HAAC01018753	0.991101	0.00889946
HAAC01019268	0.998589	0.00141057
HAAC01019686	0.999605	0.000394874
HAAC01019785	0.999824	0.000175863
HAAC01019801	0.992004	0.00799587
HAAC01020010	0.99604	0.00395959
HAAC01020049	0.977562	0.0224379
HAAC01021048	0.961368	0.0386317
HAAC01021346	0.888 0.	112
HAAC01022195	0.963069	0.0369314
HAAC01023382	0.791302	0.208698
HAAC01023849	0.996442	0.00355806
HAAC01024116	0.988248	0.0117522
HAAC01024159	0.999193	0.00080697
HAAC01024594	0.997189	0.00281105
HAAC01024738	0.998053	0.00194661
HAAC01025114	0.990437	0.00956257
HAAC01025151	0.9982 0	0.00180003
HAAC01025916	0.995188	0.00481225
HAAC01026435	0.977256	0.0227435
HAAC01028599	0.996839	0.00316057

HAAC01028684	0.995934	0.00406624
HAAC01028725	0.997421	0.0025787
HAAC01031035	0.956098	0.0439021
HAAC01031086	0.995438	0.00456234
HAAC01031175	0.998066	0.00193447
HAAC01031263	0.920271	0.0797286
HAAC01031268	0.838255	0.161745
HAAC01032009	0.8863 0	0.1137
HAAC01032556	0.821927	0.178073
HAAC01032699	0.995922	0.00407844
HAAC01033428	0.996576	0.00342416
HAAC01033995	0.821106	0.178894
HAAC01034567	0.994199	0.00580137
HAAC01036240	0.997752	0.00224788
HAAC01036268	0.967129	0.0328712
HAAC01036275	0.973147	0.0268533
HAAC01036277	0.977732	0.022268
HAAC01036342	0.938194	0.0618062
HAAC01036459	0.956997	0.0430033
HAAC01036529	0.929796	0.0702037
HAAC01038047	0.665784	0.334216
HAAC01038985	0.998892	0.00110772
HAAC01039043	0.913143	0.0868565
HAAC01039069	0.995553	0.0044474
HAAC01039245	0.990226	0.00977416
HAAC01039494	0.751448	0.248552
HAAC01039497	0.920025	0.0799749
HAAC01040826	0.905453	0.0945474
HAAC01041159	0.997909	0.00209131
HAAC01041412	0.76727	0.23273
HAAC01043641	0.927604	0.0723955
HAAC01044732	0.999083	0.000916853
HAAC01001634	0.12297	0.87703
HAAC01001983	0.15987	0.84013
HAAC01002062	0.159424	0.840576
HAAC01008310	0.31239	0.68761
HAAC01011404	0.246863	0.753137
HAAC01012748	0.288548	0.711452
HAAC01013675	0.084212	4 0.915788
HAAC01015273	0.445716	0.554284
HAAC01015703	0.612784	0.387216
HAAC01024808	0.445057	0.554943
HAAC01025551	0.813459	0.186541
HAAC01030810	0.844339	0.155661

HAAC01035083	0.05759	8 0.942409
HAAC01036065	0.243774	0.756226
HAAC01037004	0.133402	0.866598
HAAC01037954	0.139363	0.860637
HAAC01041209	0.310858	0.689142
HAAC01042509	0.5	0.5
